# Liangfang Wenjing decoction regulates endoplasmic reticulum stress-mediated apoptosis to alleviate uterine microvascular injury in cold-stressed rats

**DOI:** 10.3389/fphar.2025.1649924

**Published:** 2025-09-30

**Authors:** Tianyuan Lv, Yue Hu, Xueyan Ma, Liqun Feng, Di Wang, Xiaodan Song, Sisi Xue, Shuai Lu, Xiumei Cheng

**Affiliations:** ^1^ Graduate School, Hebei University of Chinese Medicine, Shijiazhuang, China; ^2^ Shijiazhuang Hospital of Traditional Chinese Medicine, Shijiazhuang, China; ^3^ Hebei Key Laboratory of Integrative Medicine on Liver-Kidney Patterns, Shijiazhuang, China; ^4^ Hebei Collaborative Innovation Center of Integrated Traditional and Western Medicine on Reproductive Disease, Shijiazhuang, China

**Keywords:** cold stress, Liangfang Wenjing decoction, endoplasmic reticulum stress, apoptosis, microvascular injury

## Abstract

**Background:**

Cold exposure can induce microvascular damage, which in turn contributes to various gynecological disorders. Liangfang Wenjing Decoction (LFWJD), a classical botanical drug prescription, has long been used to treat gynecological diseases caused by cold through improving microvascular injury. Nevertheless, its underlying therapeutic mechanism is still unclear. This study aimed to elucidate the effects of cold stress on uterine microvessels and to explore the pharmacological mechanisms through which LFWJD mitigates uterine microvascular injury.

**Methods:**

UPLC-MS/MS analysis was used to identify the major metabolites in LFWJD. A rat cold stress model was established using ice-water bath treatment, to investigate the mechanisms by which LFWJD ameliorates uterine microvascular injury. The study assessed several parameters, including the estrous cycle of rats, hemorheology indices, real-time blood flow in the auricle and uterine surfaces, and uterine arterial hemodynamics. Uterine histopathology and ultrastructural changes in vascular endothelial cells were observed. Serum and uterine tissue endothelial factors were quantified by ELISA, while apoptosis in uterine and vascular endothelial cells was evaluated using TUNEL staining. Western blotting and immunofluorescence were performed to detect the expression of apoptosis-related proteins, endoplasmic reticulum stress (ERS) marker proteins, and proteins associated with the IRE1α/ASK1/JNK and IRE1α/XBP1s signaling pathways.

**Results:**

Treatment with LFWJD for 21 days restored the estrous cycle in cold-stressed rats and improved uterine histopathology, as evidenced by an increased number of glands and thickening of the endometrium. LFWJD also enhanced blood perfusion in the auricle and uterine surface, regulated vascular endothelial factors (ET-1, NO, vWF, and TM), and repaired the ultrastructural integrity of endothelial cells. Moreover, it reduced apoptosis in uterine tissue and vascular endothelial cells by downregulating the expression of pro-apoptotic proteins (Bax, and cleaved caspase3) and ERS-related factors (GRP78, CHOP, XBP1s, p-IRE1α, p-ASK1, and p-JNK), while upregulating the anti-apoptotic protein Bcl-2 protein. Collectively, these pharmacological effects contributed to the alleviation of uterine microvascular injury in the uterus of cold-stressed rats.

**Conclusion:**

LFWJD may exert its pharmacological effects by inhibiting the expression of ERS-related signaling pathway proteins and alleviating apoptosis, thereby improving uterine microvascular injury. This will provide an experimental foundation for the potential clinical application of LFWJD.

## 1 Introduction

In cold environments, exposure to low temperatures can induce cold stress, a condition that triggers a series of pathological changes (Giesbrecht, 2000). The uterus is particularly sensitive to cold stress, which causes constriction of the uterine arteries, increases the pulsatility index (PI), and compromises blood supply to the endometrium (Kimura et al., 1998). Clinical studies have shown a positive correlation between cold exposure and the severity of dysmenorrhea, possibly due to reduced nitric oxide (NO) availability. Diminished NO impairs vasodilation, leading to uterine blood flow and pain (Wu et al., 2023; Liddle et al., 2022). Animal studies further demonstrate that cold tress prolongs the estrous cycle, disrupts reproductive hormone levels, and alters uterine morphology (Xu et al., 2018). Additionally, cold exposure reduces NO levels and impairs uterine blood circulation (Rennie et al., 2015). Previous findings indicate that cold stress also disrupts the balance between the vasoconstrictor endothelin-1 (ET-1) and the vasodilator NO, thereby impairing microvascular endothelial function and reducing uterine microcirculatory perfusion (Wang et al., 2020a).

Vascular endothelial cells play a central role in maintaining vascular homeostasis and regulating microcirculation (Xu et al., 2021). Cold stress increases the release of ET-1 while reducing the release of NO from endothelial cells, creating an imbalance between vasoconstrictive and vasodilatory factors. This imbalance impairs vasodilation, elevates vascular resistance, and triggers excessive vasoconstriction, ultimately resulting in insufficient blood supply and endothelial dysfunction (Eglin et al., 2023; Valtonen et al., 2020). Endothelial dysfunction is recognized as one of the key mechanisms underlying cold-induced uterine microvascular injury. Thus, investigating the effects of cold stress on vascular endothelial function is essential for developing strategies to prevent and treat cold-related uterine diseases. Endothelial cell apoptosis is considered a critical mechanism contributing to vascular endothelial dysfunction (Li K. et al., 2025). Endoplasmic reticulum stress (ERS) plays a pivotal role in regulating this process (Burgos et al., 2019; Li and Sun, 2024). ERS is a pathological state induced by the accumulation of unfolded proteins when endoplasmic reticulum function is disrupted. Under such conditions, the cell activates the unfolded protein response (UPR) to restore homeostasis; however, if ERS is excessive or prolonged, apoptosis may be triggered (Wan et al., 2022). Modulating ERS can therefore attenuate endothelial cell apoptosis and preserve endothelial function. Among the ERS-mediated apoptotic pathways, the IRE1α/XBP1s and IRE1α/ASK1/JNK signaling cascades are the most clearly defined and extensively studied.

Liangfang Wenjing Decoction (LFWJD) is a classical traditional Chinese medicine (TCM) prescription recorded in Practical Prescriptions for Women. In TCM theory, cold is characterized by induction and stagnation, leading to the contraction and meridians and veins, as well as blood coagulation (Jung et al., 2018). LFWJD is particularly effective in warming the meridians and promoting blood circulation. It is regarded as a classical formula for treating gynecological disorders associated with cold-induced coagulation and blood stasis (Zuo et al., 2022) and is widely used in the clinical management of conditions such as primary dysmenorrhea, endometriosis, uterine polyps, and menstrual disorders caused by cold (Li XL. et al., 2025; Gao et al., 2017). Previous clinical studies have demonstrated that LFWJD can regulate ET-1 and NO levels in patients with cold-induced menstrual disorders, thereby improving vascular diastolic function and alleviating hypercoagulability. These pharmacological effects contribute to the relief of symptoms such as hypomenorrhea, dysmenorrhea, and delayed menstruation (LU et al., 2020). Animal experiments further confirm that LFWJD improves uterine microcirculatory disturbances and microvascular damage in cold-stressed rats, while enhancing vascular endothelial function (Xing et al., 2024; Wang et al., 2020b). Nevertheless, the precise mechanisms through which LFWJD mitigates uterine microvascular injury under cold stress remain unclear and warrant further exploration.

4-Phenylbutyric acid (4-PBA) is a specific inhibitor of ERS that functions as a molecular chaperone, reducing the aggregation of misfolded and unfolded proteins under stress conditions (Zhou et al., 2024; Wang and Tang, 2020). Numerous studies have shown that 4-PBA can alleviate ERS, suppress cell apoptosis, and improve tissue and organ function (Chen et al., 2022). In this study, 4-PBA was used as an intervention in cold-stressed rats to serve as a mechanistic tool for validating the involvement of the ERS pathway.

Therefore, this study established a rat cold stress model to investigate the effects of cold stress on uterine microvascular injury. Furthermore, we explored the mechanisms by which LFWJD regulates ERS to attenuate apoptosis and ameliorate uterine microvascular damage in cold-stressed rats. This will provide an experimental foundation for the therapeutic application of LFWJD in the treatment of cold-induced gynecological disorders.

## 2 Materials and methods

### 2.1 Drugs preparation and administration

The following drugs were used in this study: 4-PBA for injection (HY A0281, MedChemExpress, Shanghai, China); and LFWJD formula granules, which contain 0.8 g Cinnamomi Cortex [Lauraceae; *Cinnamomum verum* J.Presl], Batch No. 21062921; 1.5 g Angelicae Sinensis Radix [Apiaceae; *Angelica sinensis* (Oliv.) Diels], Batch No. 25041061; 1.1 g Chuanxiong Rhizoma [Apiaceae; *Ligusticum chuanxiong* Hort], Batch No. 25010461; 0.5 g Paeoniae Radix Alba [Paeoniaceae; *Paeonia lactiflora* Pall.], Batch No. 25020974; 0.3 g Curcumae Rhizoma [Zingiberaceae; *Curcuma zedoaria* (Christm.) Roscoe], Batch No. 24070971; 0.6 g Moutan Cortex [Paeoniaceae; *Paeonia suffruticosa* Andr.], Batch No. 25011861; 2.5 g Achyranthis Bidentatae Radix [Amaranthaceae; *Achyranthes bidentata* Blume], Batch No. 24101161; 1.8 g Ginseng Radix et Rhizoma [Araliaceae; *Panax ginseng* C. A. Mey.], Batch No. 25030272; and 1.7 g Glycyrrhizae Radix et Rhizoma [Fabaceae; *Glycyrrhiza glabra* L.], Batch No. 25042571. The LFWJD formula granules were purchased from Shineway Pharmaceutical Co., Ltd. (SC12713011100083, Shijiazhuang, China). The purchased LFWJD formula granules are dissolved in double-distilled water for animal experiments. The plant names were verified with MPNS (http://mpns.kew.org). UHPLC-MS/MS analysis was performed to characterize the metabolites of LFWJD (Heinrich et al., 2020).

### 2.2 Animal modeling and grouping

Fifty female SD rats (SPF, 7 weeks old, 200 ± 10 g) were obtained from Beijing Vital River Laboratory Animal Technology Co., Ltd. (SCXK, 2021-0011, Beijing, China). The rats were maintained at 20 °C–24 °C, a humidity of 40%–60%, and a 12 h/12 h light-dark cycle and were provided free access to water and food. The rats were adaptively fed for 1 w and then randomly divided into a control group, model group, low-dose LFWJD administration group (LFWJD-L group), high-dose WJD administration group (LFWJD-H group), and 4-PBA group, with 10 rats in each group (Cao et al., 2023).

The control group underwent no experimental intervention and remained undisturbed throughout the experiment, while the other groups were subjected to cold stress using the ice-water bath method. Cold stress was simulated by immersing each rat in ice water (0 °C-1 °C) daily at 9:00 a.m., with the water level maintained at the neck to ensure unobstructed breathing. Each immersion lasted for 20 min. After cold exposure, the rats were immediately removed, gently dried with a towel, and further dried with a hair dryer to prevent hypothermia-related mortality. This procedure was performed for 21 consecutive days. This protocol was based on the Cheng Xiumei pharmacological method (Wang et al., 2020a; Cheng et al., 2014; Sun et al., 2025), which is widely used in pharmacological research to mimic the physiological effects of cold stress in women.

LFWJD-H and LFWJD-L groups were gavaged LFWJD granules (0.47 g/kg, 0.93 g/kg) for 21 consecutive days. The equivalent rat dosages were calculated based on the original prescription recorded in Furen Daquan Liangfang, modern clinical dosage references, and body surface area conversion (coefficient 6.3) (Cao et al., 2023). 4-PBA was dissolved in saline to prepare the treatment solution. An equimolar quantity of sodium hydroxide was added to 4-PBA and titrated, resulting in an injectable formulation with a pH of 7.4 and a 4-PBA concentration of 20 mg/mL. Following 7 days of cold stress induction, rats in the 4-PBA group received intraperitoneal injections of this solution at 100 mg/kg/day for 14 days continued concomitantly with the remaining duration of the cold stress protocol (Figure 2A).

### 2.3 Estrous cycle

Exfoliated cells were stained with methylene blue solution (G1300, Solarbio, China) for 30 min, after which cell morphology and estrous cycle stages were examined under a microscope (Nikon Eclipse E100, Nikon, Japan).

### 2.4 Uterine artery blood flow parameters

Rats were anesthetized with 2% isoflurane (R510-22, RWD Life Science Co., Ltd., China) and placed in a supine position on the test table. After abdominal hair removal, the ultrasound probe was positioned in close contact with the skin, and uterine arterial hemodynamic parameters were measured using a VisualSonics VEVO 2100 ultrasound imaging system (VisualSonics, Canada).

### 2.5 Hemorheology index and regional blood flow

Heparinized blood samples were analyzed for hemorheological parameters using an automatic hemorheology instrument (LBY-N75008 Beijing Precision Instrument Co., Ltd.). Uterine tissues were fully exposed, and real-time blood flow monitoring and image acquisition were performed using the Moor FLPI-2 Scattered Spot Flow Imaging System (Moore Instruments Co., Ltd.).

### 2.6 Serum and uterine tissue vascular endothelial factor assays

Blood and uterine tissue homogenates were centrifuged at 3000 r/min for 15 min at 4 °C using a high-speed refrigerated centrifuge (Thermo Fisher Scientific Co., Ltd., China). The resulting supernatants were collected, and the levels of ET-1, NO, von Willebrand factor (vWF), and thrombomodulin (TM) in serum and uterine tissue were quantified by ELISA. Measurements were performed using enzyme-linked immunosorbent assay kits (Bioswamp, China; item nos. RA20560, BTK006, RA20658, and RA20453) according to the manufacturer’s instructions.

### 2.7 Morphological observation

Uterine tissues were fixed in 4% paraformaldehyde followed by dehydration, transparentizing, wax immersion, and embedding, tissues were cut into 4 μm sections and placed on slide glass. They were stained with HE (G1003, Servicebio, China). Images were captured using a microscope.

### 2.8 Ultrastructure of microvascular endothelial cells

Uterine tissues were initially fixed in 2.5% glutaraldehyde solution (G1102, Servicebio, China). Ultrathin sections (60 nm) were then prepared and stained with 2% uranyl acetate followed by 2.6% lead citrate. The ultrastructure of uterine microvascular endothelial cells was examined using transmission electron microscopy (TEM) (Thermo Fisher Scientific Co., Ltd., China).

### 2.9 TUNEL analysis

A TUNEL assay (G1501, Servicebio, China) was utilized to detect apoptotic cells in uterine tissue sections. The sections were incubated overnight at 4 °C with a primary antibody against CD34 (1:300, Ab81289, Abcam, United States), washed with PBS, and then incubated with a secondary antibody in the dark for 50 min at room temperature. After another PBS wash, the sections were counterstained with DAPI (G1012, Servicebio, China) for 10 min at room temperature in the dark. Fluorescence images were observed under a fluorescence microscope (Nikon Eclipse C1, Nikon, Japan). TUNEL-positive cells appeared green, CD34-positive cells red, and double-positive (TUNEL/CD34) cells yellow. Green fluorescence indicated apoptotic cells, and yellow fluorescence represented apoptotic vascular endothelial cells. Quantitative analysis was performed by measuring staining intensity, expressed as average optical density, using ImageJ software (version 1.53).

### 2.10 Immunofluorescence staining

Uterine paraffin sections were blocked with 3% BSA (Sigma, United States) at 37 °C for 30 min and then incubated overnight at 4 °C with primary antibodies: GRP78 (1:300, 11587-1-AP, Proteintech, United States), CHOP (1:300, YM3668, Immunoway, United States), Bax (1:200, GB114122, Servicebio, China), Bcl-2 (1:200, 26593-1-AP, Proteintech, United States), and the endothelial cell marker rat anti-CD34 (1:300). After washing, sections were incubated with fluorescent secondary antibodies at room temperature for 2 hours in the dark. Nuclei were restrained with DAPI. The stained specimens were observed by fluorescence microscope.

### 2.11 Western blotting

Uterine tissues were lysed in RIPA buffer containing phosphatase and protease inhibitors (G2002, Servicebio, China). Protein concentrations were calculated using a BCA protein assay kit (G2026, Servicebio, China). Equal amounts of protein were separated by SDS‒PAGE and transferred onto PVDF membranes. After blocking with 5% BSA at room temperature for 2 h, the membranes were incubated overnight at 4 °C with the following primary antibodies: GRP78 (1:5000), CHOP (1:1000), Bax (1:1000), Bcl-2 (1:2000), caspase-3 (1:800, 9662, CST, United States), eNOS (1:1000, 27120-1-AP, Proteintech, United States), p-eNOS (1:1000, 9571, CST, United States), XBP1s (1:1000, 40435, CST, United States), IRE1α (1:1000, 3924, CST, United States), p-IRE1α(1:1000, PA1-16927, Thermo, United States), ASK1 (1:1000, 8662, CST, United States), p-ASK1 (1:1000, MA5-36973, Thermo, United States), JNK (1:1000, 9252, CST, United States), p-JNK (1:1000, 4668, CST, United States), GAPDH (1:10,000, 60004-1-Ig, Proteintech, United States), and β-tubulin (1:5000, 66240-1-Ig, Proteintech, United States). Following incubation, membranes were washed and incubated with HRP-conjugated secondary antibodies in TBST for 30 min at room temperature. Immunoreactive bands were visualized and analyzed on an AIWBwellTM Imaging System (Servicebio, China).

### 2.12 Statistical analyses

All experiments were performed at least three times. Data are presented as the mean ± standard deviation (SD). GraphPad Prism 8 was used to plot the data. One-way ANOVA was used to analyze the data between multiple groups when the data met normality criteria. Where variances were homogeneous, Tukey’s test was utilized for *post hoc* comparisons. Tamhane’s T2 test was used when a normal distribution was not satisfied, or the variance was uneven. Differences were considered statistically significant when *P < 0.05*.

## 3 Results

### 3.1 Metabolites of LFWJD

**FIGURE 1:**
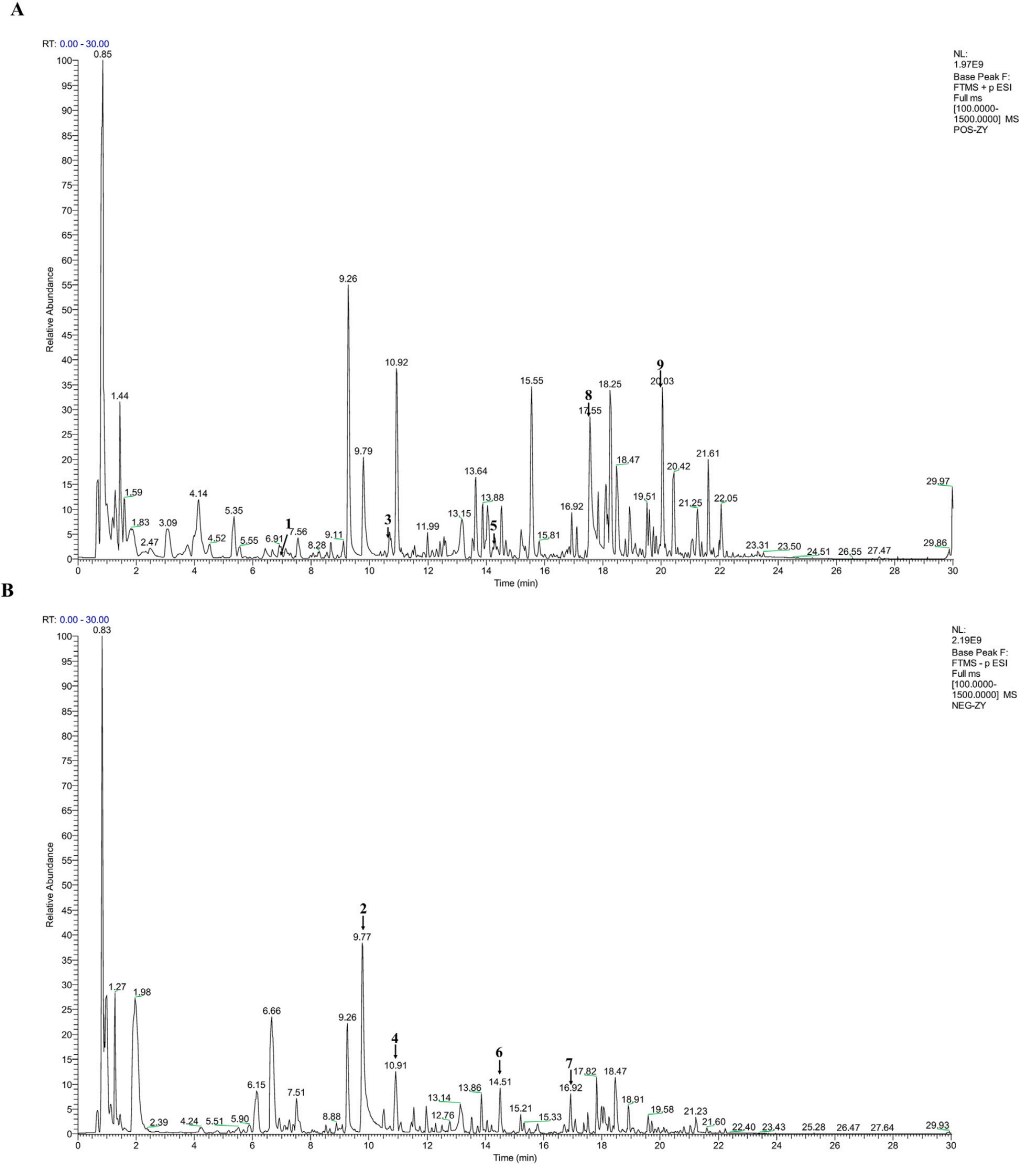
Qualitative analysis of LFWJD by UHPLC-MS/MS. **(A)** BPI plot of LFWJD in the positive ion mode. **(B)** BPI plot of LFWJD in the negative ion mode. (1) Catechin; (2) Paeoniflorin; (3) Ferulic acid; (4) Liquiritin; (5) Senkyunolide H; (6) Liquiritigenin; (7) Benzoylpaeoniflorin; (8) Paeonol; (9) Curcumenol.

UHPLC-MS/MS was employed to characterize the metabolites of LFWJD. The collected data were qualitatively analyzed using Progenesis QI 3.0 software, and the metabolites were identified by combining them with the reference substance database, PubChem database (pubchem.ncbi.nlm.nih.gov), TCMSP database (old.tcmsp-e.com/tcmsp.php) and related literature (Li XL. et al., 2025). We found that the primary metabolites of this formulation were catechin, paeoniflorin, ferulic acid, liquiritin, senkyunolide H, liquiritigenin, benzoylpaeoniflorin, paeonol, and curcumenol. The base peak io (BPI) plots and comprehensive details regarding metabolites are provided (Figure 1; Supplementary Table S1).

### 3.2 Pharmacological effects of LFWJD on estrous cycle and uterine morphology of cold-stressed rats

**FIGURE 2:**
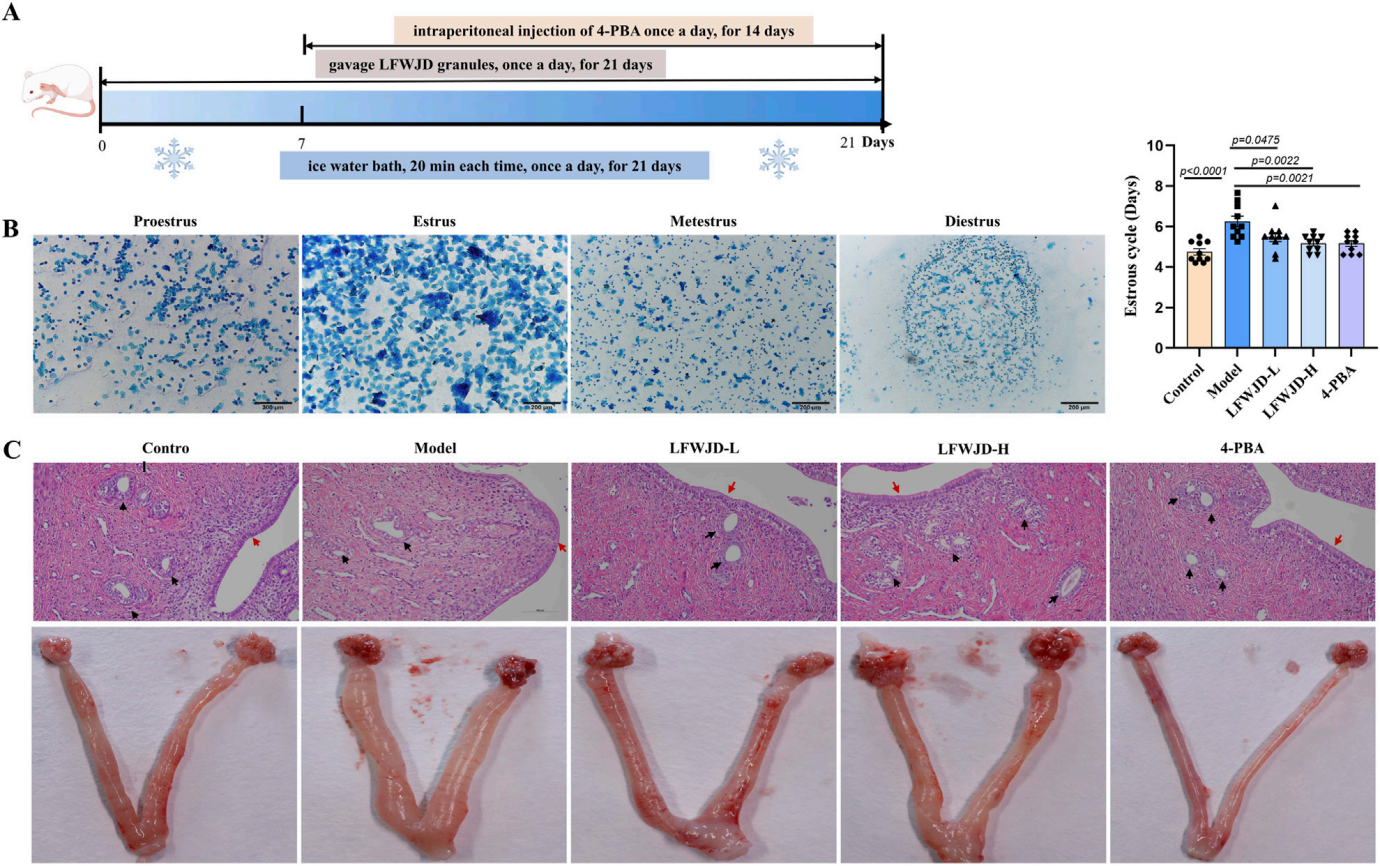
LFWJD improves the general condition of cold-stressed rats. **(A)** Schematic of the experimental procedure for cold-stressed rats and LFWJD intervention. **(B)** Representative microscopy images (×200 magnification; scale bar = 200 µm) of different stages of the estrous and quantitative data analysis of the estrous cycle. Data are presented as mean ± SD (n = 10). Statistical significance was determined by one-way ANOVA followed by Tukey’s *post hoc* test. **(C)** Representative images of H&E-stained uterine sections (×200 magnification; scale bar = 100 µm). Black arrows point to glandular structures; red arrows highlight the endometrium.

The estrous cycle is an important expression of reproductive function. The morphology of vaginal exfoliated cells varies across different estrous cycles. The estrus was characterized by the anucleate keratinized epithelial cells. Metestrus was marked by a balanced mix of non-nucleated keratinized epithelial cells, leukocytes, and nucleated epithelial cells. Diestrus was dominated by numerous leukocytes. Proestrus was dominated by numerous nucleated epithelial cells. In the control group, the estrus cycle was regular for 4–5 days. Rats in the model group had a prolonged estrous cycle (*P < 0.05*), the estrous cycle was gradually restored after LFWJD and 4-PBA interventions (*P < 0.05*) (Figure 2B). The uterine pathologic morphology was different in each group of rats. The model group had oedema of uterine tissues, thinning of endometrial epithelial cells, scanty glands and fewer stromal cells in the lamina propria. After LFWJD and 4-PBA interventions, oedema of uterine tissues was reduced, glands were increased, and endometrium was thickened (Figure 2C).

### 3.3 Pharmacological effects of LFWJD on blood flow status in cold-stressed rats

Blood hemorheology is an indication of the state of the blood. Compared with the control group, the hemorheological indices in the model group, whole blood reduced viscosity, and the erythrocyte aggregation index, were increased (*P < 0.05*). The hemorheological indexes decreased after the intervention of LFWJD and 4-PBA (*P < 0.05*) (Supplementary Table S2).

**FIGURE 3:**
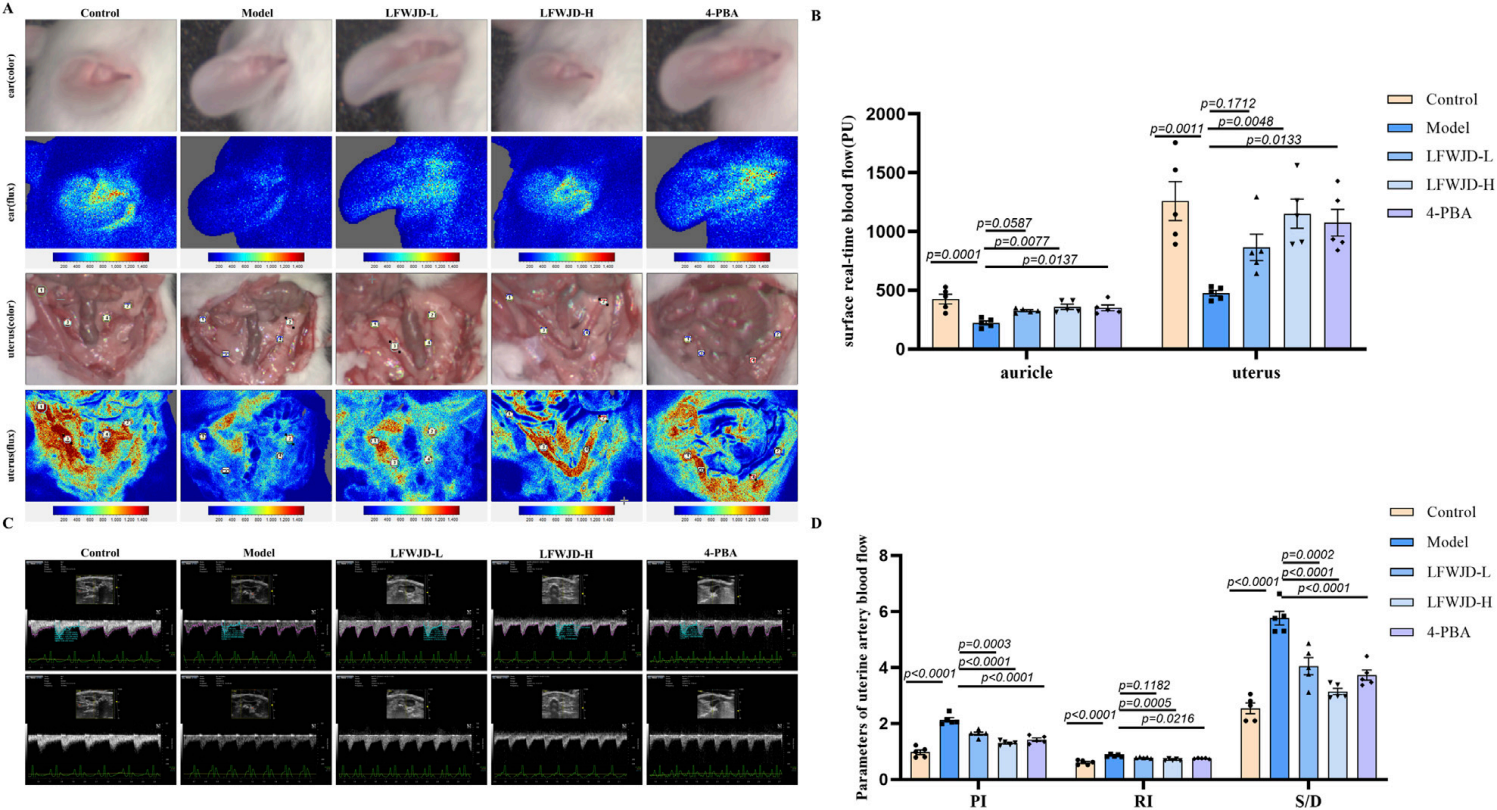
Auricular and uterine microcirculatory blood flow and uterine hemodynamic parameters. **(A)** Real-time *in vivo* monitoring of microvascular blood flow on the surfaces of the rat auricle and uterus. **(B)** Quantitative analysis of auricular and uterine surface blood flow. Data are presented as mean ± SD (n = 4). Statistical significance was determined by one-way ANOVA followed by Tukey’s *post hoc* test. **(C)** Blood flow dynamics of uterine arteries, including PI, RI, and S/D, were measured using ultrasound imaging system. **(D)** Quantitative analysis of uterine artery blood flow parameters. Data are presented as mean ± SD (n = 5). Statistical significance was determined by one-way ANOVA followed by Tukey’s *post hoc* test.

Cold stress can lead to changes in microcirculatory blood flow. Compared with the control group, auricular and uterine microcirculatory blood flow were decreased in cold-stressed rats (*P < 0.05*). Relative to the model group, the LFWJD-L group showed a significant increase in auricular microcirculatory blood perfusion (P < 0.05), while the LFWJD-H and 4-PBA groups exhibited increased blood flow in both auricular and uterine surface microcirculation (*P < 0.05*). These results suggest that LFWJD can ameliorate local microcirculatory blood flow impairment in cold-stressed rats (Figures 3A,B).

A higher value of PI means lower blood flow in the blood vessel, a higher value of Resistance Index (RI) means higher resistance to blood flow, and a higher value of Systolic/Diastolic ratio (S/D) means slower blood flow. Compared with the control group, PI, RI, and S/D of uterine arteries were elevated in the model group (*P < 0.05*). The PI, RI, and S/D of uterine arteries were reduced after LFWJD and 4-PBA intervention, suggesting that LFWJD can improve uterine artery hemodynamic parameters (*P < 0.05*). Figures 3C,D).

### 3.4 Pharmacological effects of LFWJD on endothelial function in cold-stressed rats

**FIGURE 4:**
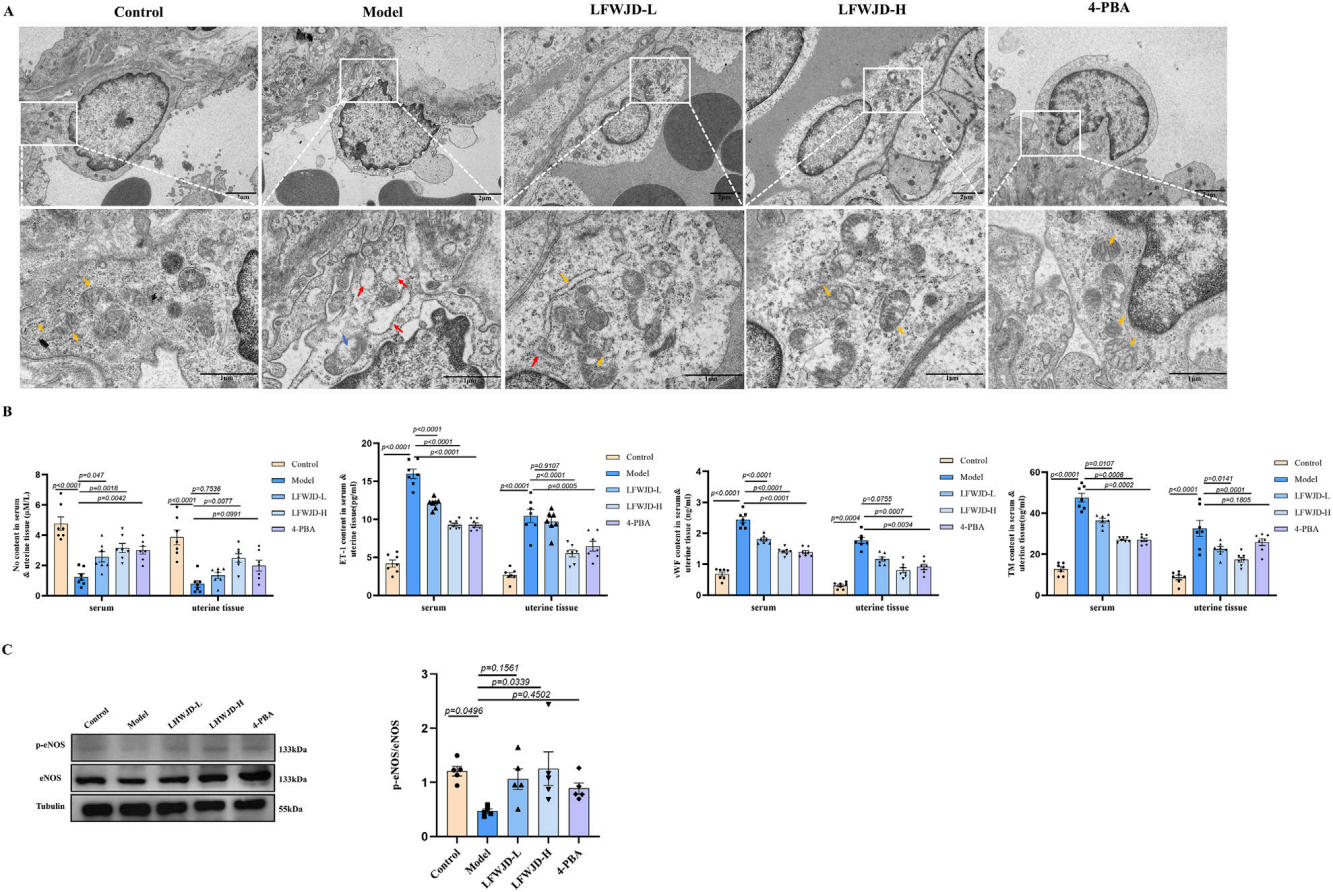
Pharmacological effects of LFWJD on endothelial function in cold-stressed rats. **(A)** TEM reveals the ultrastructural features of uterine vascular endothelial cells (15.0k × magnification; scale bar = 100 µm). Yellow arrows indicate normal organelles; red arrows show swollen and dilated endoplasmic reticulum; blue arrows denote vacuolated mitochondria. **(B)** Quantification of ET-1, NO, TM, and vWF levels in serum and uterine tissues. Data are presented as mean ± SD (n = 7). **(C)** Analysis of eNOS and p-eNOS expression by Western blot (loading control: Tubulin). Protein band densities were quantified with ImageJ, and the p-eNOS/eNOS ratio was calculated using GraphPad Prism 8. Data are presented as mean ± SD (n = 5). Statistical significance was determined by one-way ANOVA followed by Tukey’s *post hoc* test.

Ultrastructural revealed that vascular endothelial cells in the control group were rich in organelles, with normal endoplasmic reticulum and mitochondrial morphology. In the model group, the number of organelles was reduced, the endoplasmic reticulum appeared swollen and dilated, and mitochondrial cristae were broken and vacuolated. Treatment with LFWJD and 4-PBA ameliorated mitochondrial and endoplasmic reticulum damage in endothelial cells (Figure 4A).

ET-1 and NO are key regulators of vascular dilation and contraction, while TM and vWF serve as markers of endothelial injury. In rats with cold stress, NO content decreases, while ET-1, VWF, and TM levels increase (*P < 0.05*). Following LFWJD and 4-PBA treatment, NO levels were increased, whereas ET-1, vWF, and TM levels were reduced in both serum and uterine tissues (*P < 0.05*) (Figure 3B). eNOS is a critical enzyme for NO production, and p-eNOS represents the active state. The p-eNOS/eNOS ratio was increased in the LFWJD-H group, indicating that LFWJD improves vascular endothelial function (*P < 0.05*) (Figure 4C).

### 3.5 Pharmacological effects of LFWJD on apoptosis in uterine tissues

**FIGURE 5:**
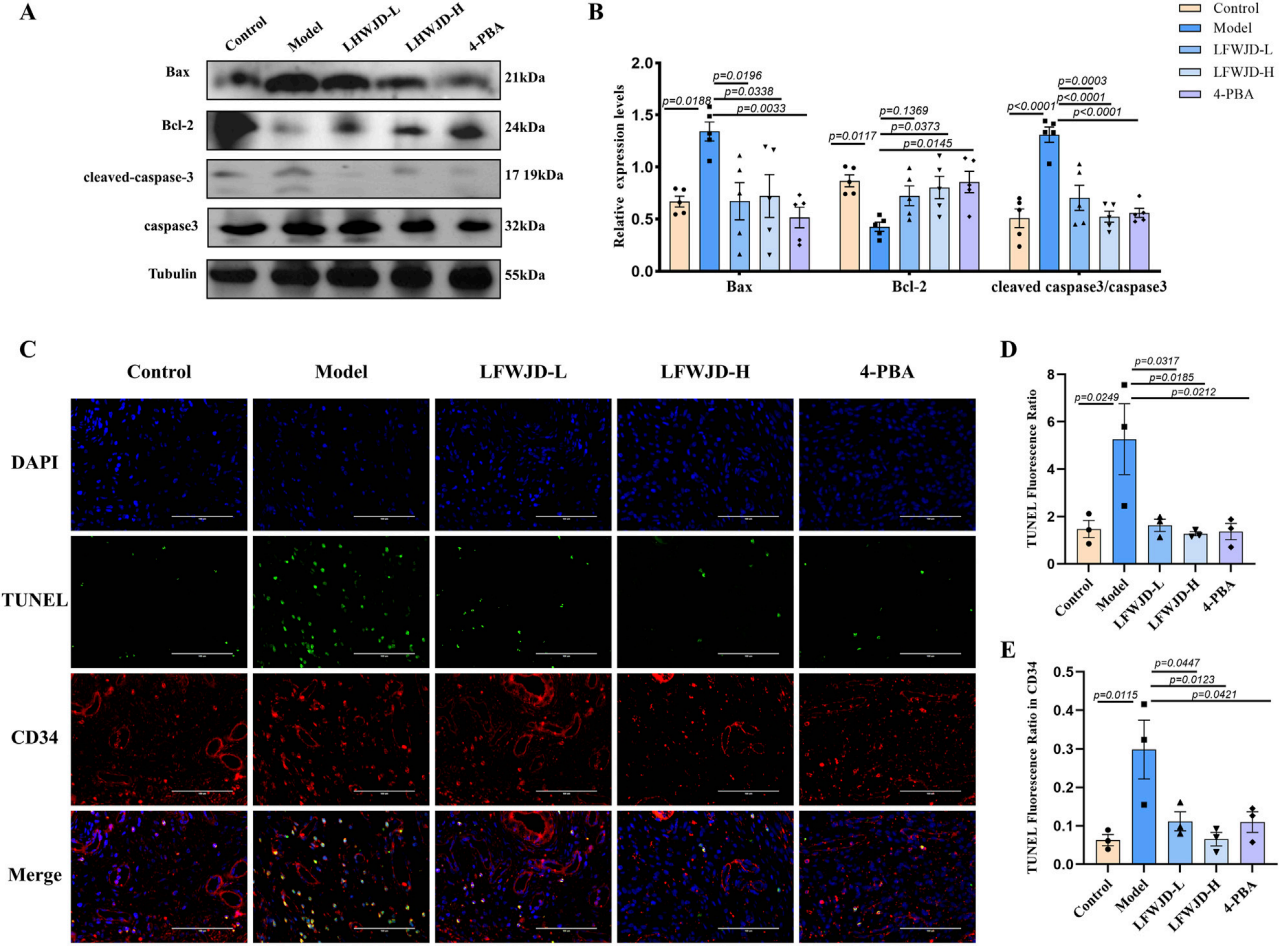
Pharmacological effects of LFWJD on apoptosis in uterine tissues. **(A)** Apoptosis-related proteins (Bax, Bcl-2, cleaved caspase-3, and caspase-3) were analyzed by Western blot, with Tubulin serving as a loading control. Protein band densities were quantified using ImageJ software. **(B)** The expression levels of Bax, Bcl-2, and the cleaved caspase-3 to caspase-3 ratio in uterine tissues were quantified using GraphPad Prism 8. Data are presented as mean ± SD (n = 5). Statistical significance was determined by one-way ANOVA followed by Tukey’s *post hoc* test. **(C)** Representative fluorescence microscopy images (×400 magnification; scale bar = 100 µm) of TUNEL staining showing apoptosis in uterine tissues and CD34-labeled vascular endothelial cells. **(D,E)** The TUNEL-positive fluorescence ratio was quantified in both the entire tissue and CD34-positive areas. Data are presented as mean ± SD (n = 3). Statistical significance was determined by one-way ANOVA followed by Tukey’s *post hoc* test.

Western blot results showed that in the model group, the expression of the pro-apoptotic protein Bax and the ratio of cleaved-caspase3 to caspase3 were increased, while the expression of the anti-apoptotic protein Bcl-2 was decreased. Compared with the model group, Bax protein expression and cleaved-caspase3/caspase3 decreased, and Bcl-2 protein expression increased in the LFWJD-L, LFWJD-H, and 4-PBA groups (*P < 0.05*) (Figures 5A,B). TUNEL results indicated that the apoptotic rates in uterine tissue and in CD34-labeled vascular endothelial cells were increased in cold-stress rats (*P < 0.05*). Following LFWJD and 4-PBA intervention, these apoptotic rates were significantly reduced (*P < 0.05*) (Figures 5C–E).

### 3.6 Pharmacological effects of LFWJD on Bax and Bcl-2 expression in uterine microvascular endothelial cells

**FIGURE 6:**
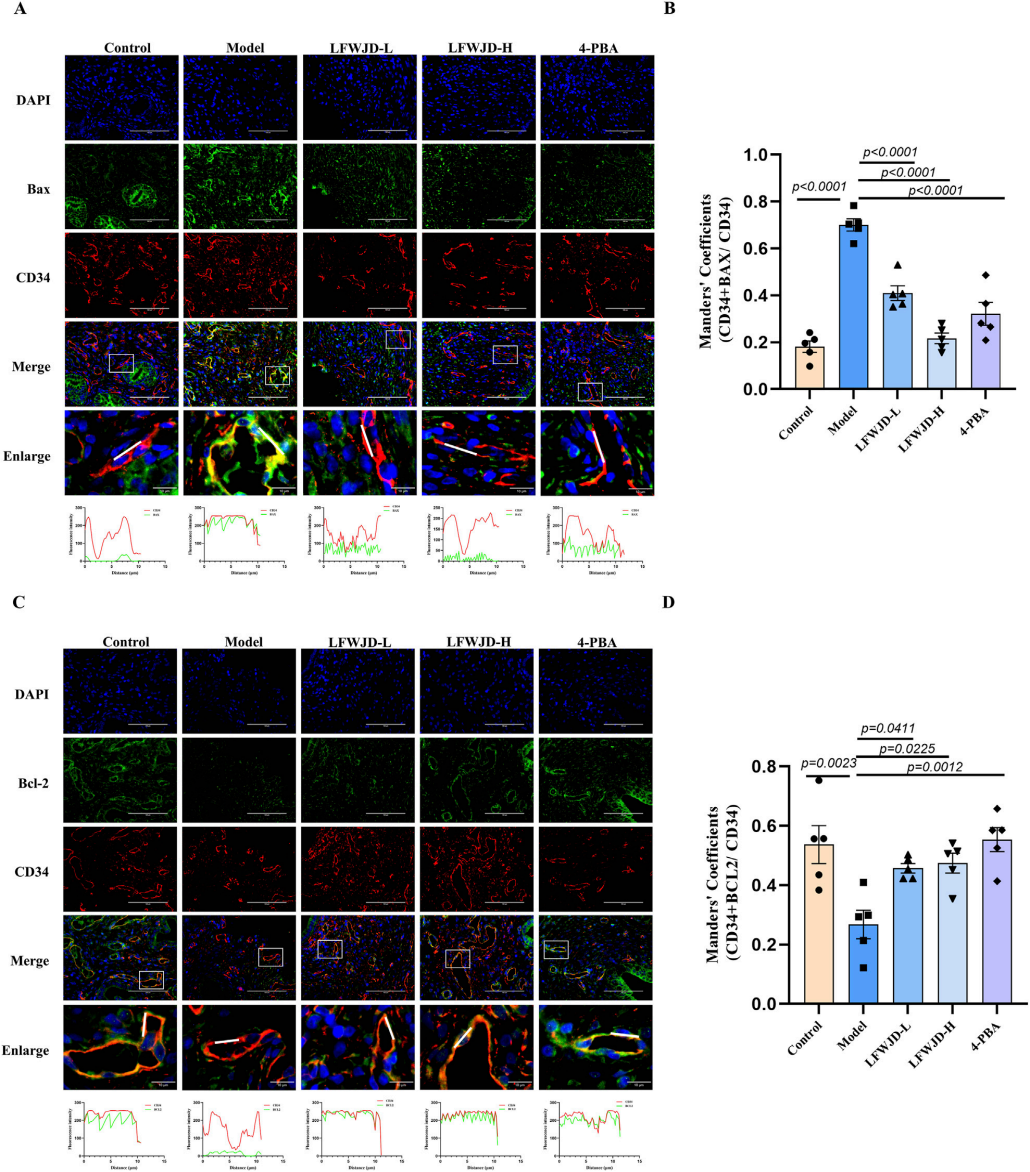
Pharmacological effects of LFWJD on Bax and Bcl-2 expression in uterine microvascular endothelial cells. **(A,C)** Representative fluorescence microscopy images (×400 magnification; scale bar = 100 µm) of Bax and Bcl-2 expression in CD34-labeled uterine microvascular endothelial cells. **(B,D)** Quantitative analysis of Bax and Bcl-2 fluorescence intensity in CD34-positive areas was performed using ImageJ and GraphPad Prism 8 software. Data are presented as mean ± SD (n = 5). Statistical significance was determined by one-way ANOVA followed by Tukey’s *post hoc* test.

Uterine microvascular endothelial cells were labeled with CD34. In the model group, the co-localization of CD34 (red) and Bax (green) was increased (*P < 0.05*) and the co-localization of CD34 (red) and Bcl-2 (green) was decreased (*P < 0.05*). This indicates that cold stress causes apoptosis in microvascular endothelial cells, leading to microvascular damage. After the intervention of LFWJD and 4-PBA, the co-localization of CD34 with Bax decreased (*P < 0.05*) and that of CD34 with Bcl-2 increased (*P < 0.05*). This suggests that LFWJD reduces the apoptosis of endothelial cells in rats under cold stress and indirectly indicates that LFWJD protects against microvascular damage (Figures 6A–D).

### 3.7 Pharmacological effects of LFWJD on ERS and related pathway proteins

**FIGURE 7:**
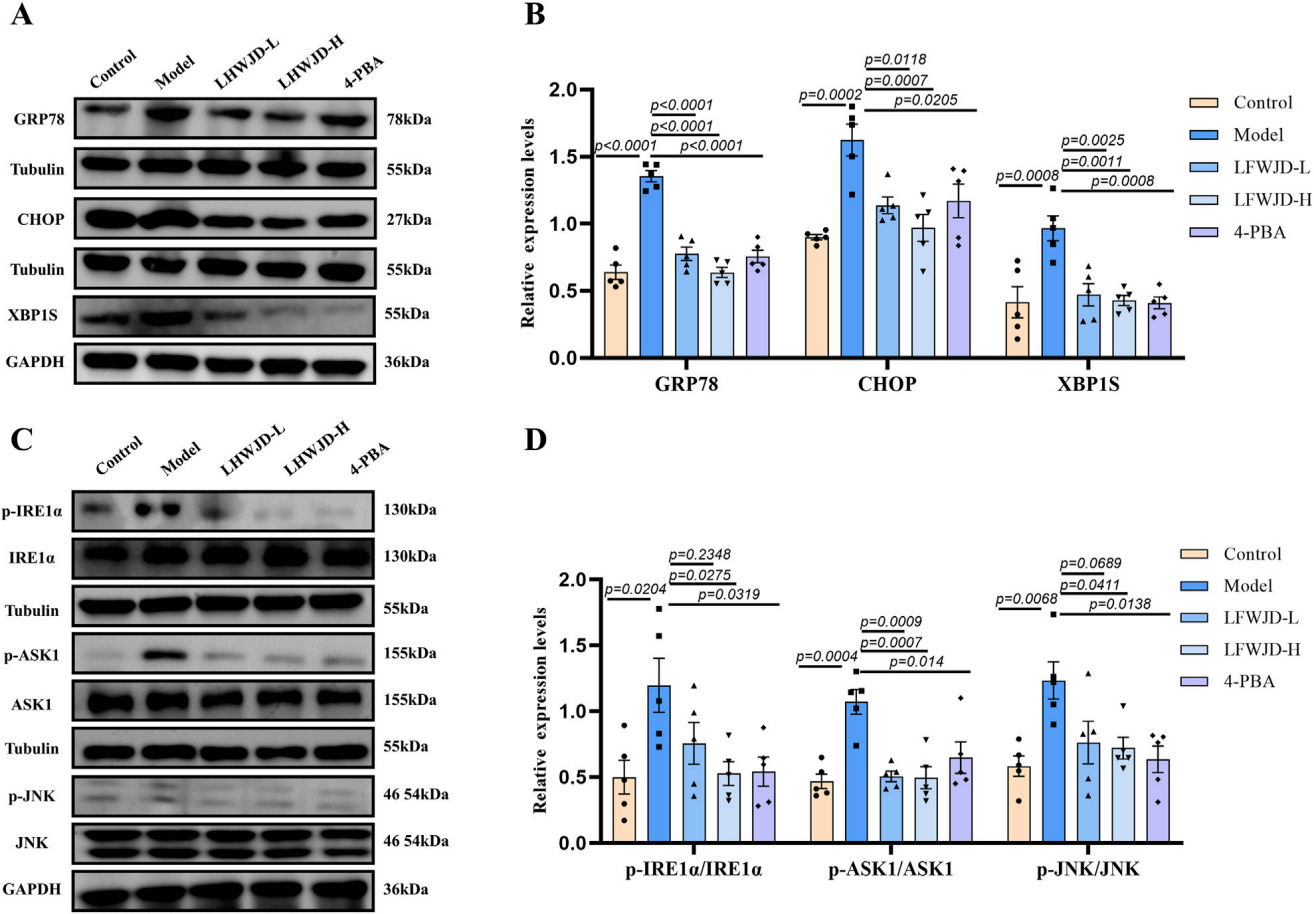
LFWJD reduces the expression of endoplasmic reticulum stress-related proteins in rats under cold stress. **(A,C)** The protein levels of GRP78, CHOP, and XBP1s related to ERS, as well as the phosphorylated and total levels of IRE1α, ASK1, and JNK, were determined using Western blot, with Tubulin and GAPDH serving as internal controls. The densitometry of indicated proteins was quantified using the ImageJ software. **(B,D)** Quantitative analysis of GRP78, CHOP, XBP1s, and the p-IRE1α to IRE1α, p-ASK1 to ASK1, and p-JNK to JNK ratios in uterine tissues was performed using GraphPad Prism 8. Data are presented as mean ± SD (n = 5). Statistical significance was determined by one-way ANOVA followed by Tukey’s *post hoc* test.

Increases in GRP78 and CHOP protein levels are hallmarks of ERS. GRP78 and CHOP expression were increased in the model group (*P < 0.05*). Compared with the model group, their expression was reduced in the LFWJD-L, LFWJD-H, and 4-PBA groups (*P < 0.05*) (Figures 7A,B). To further assess whether the IRE1α/XBP1s and IRE1α/ASK1/JNK signaling pathways were activated in cold-stressed rats, we examined the expression of related ERS proteins. The levels of XBP1s, p-IRE1α/IRE1α, p-ASK1/ASK1, and p-JNK/JNK were increased in the model group (*P < 0.05*). Compared with the model group, The levels of XBP1s, p-IRE1α/IRE1α, p-ASK1/ASK1, and p-JNK/JNK were decreased after the intervention of LFWJD and 4-PBA (*P < 0.05*) (Figures 7C,D).

### 3.8 Pharmacological effects of LFWJD on GRP78 and CHOP expression in uterine microvascular endothelial cells

**FIGURE 8:**
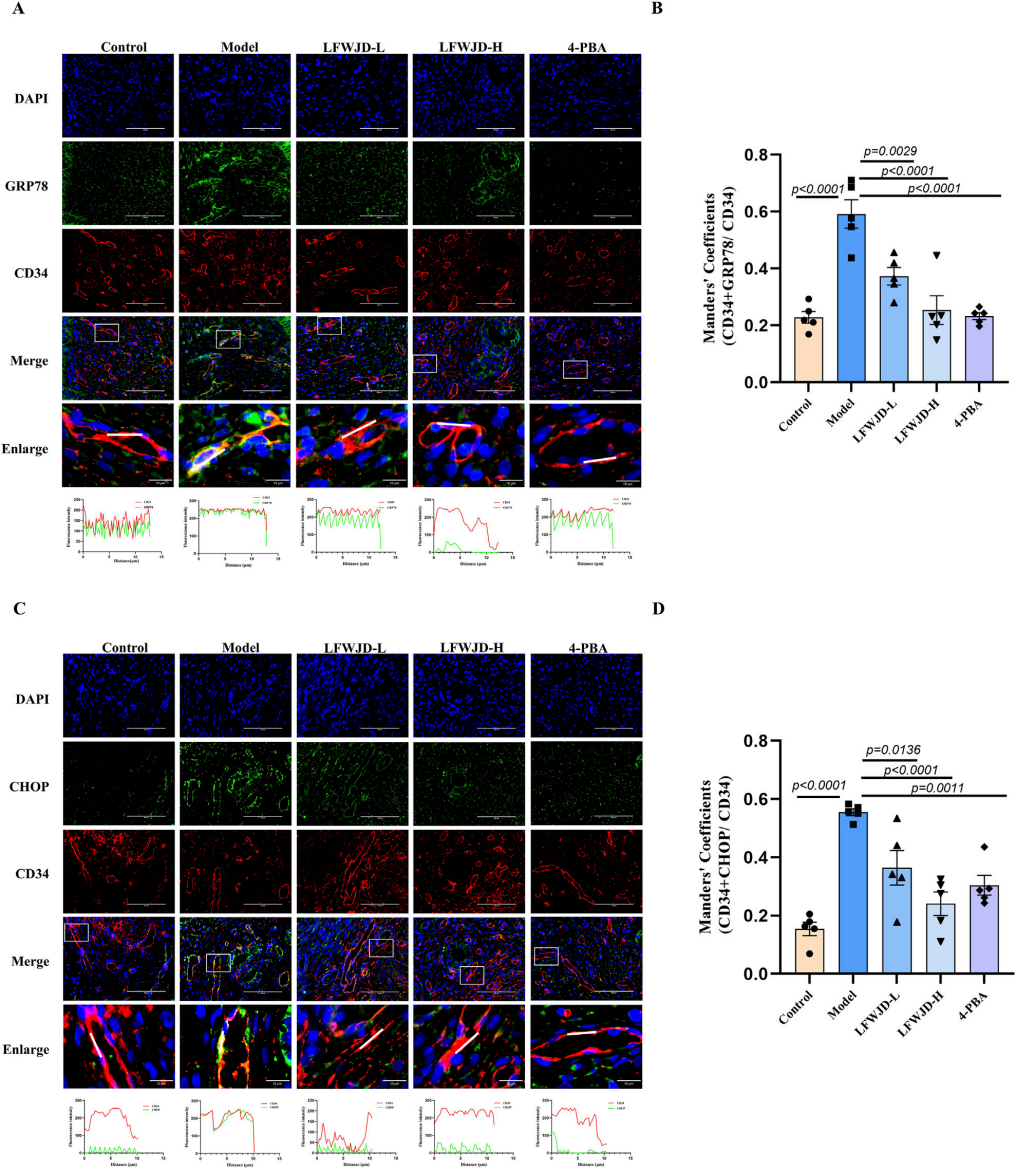
Pharmacological effects of LFWJD on GRP78 and CHOP expression in uterine microvascular endothelial cells. **(A,C)** Representative fluorescence microscopy images (×400 magnification; scale bar = 100 µm) of GRP78 and CHOP expression in CD34-labeled uterine microvascular endothelial cells. **(B,D)** Quantitative analysis of GRP78 and CHOP fluorescence intensity in CD34-positive areas was performed using ImageJ and GraphPad Prism 8 software. Data are presented as mean ± SD (n = 5). Statistical significance was determined by one-way ANOVA followed by Tukey’s *post hoc* test.

Co-localization of GRP78 (green) and CHOP (green) with CD34 were increased in the model group (*P < 0.05*), which indicates that cold can induce ERS in microvascular endothelial cells. Compared with the model group, the co-localization of GRP78 and CHOP with CD34 were reduced after the intervention of LFWJD and 4-PBA. This suggests that LFWJD can alleviate ERS induced by cold in microvascular endothelial cells, thereby protecting microvessels (*P < 0.05*) (Figures 8A–D).

## 4 Discussion

Cold exposure is a major contributing factor to gynecological disorders, and recent studies have confirmed its detrimental effects on female reproductive function (Espinoza et al., 2023). Hypothermic stimulation induces peripheral vasoconstriction, increases blood viscosity, and elevates vascular resistance (Wang et al., 2024). Studies have shown that increased blood viscosity is closely related to blood rheological characteristics (Ning et al., 2012; Alba et al., 2019). In this study, cold stress resulted in prolonged estrous cycles, uterine edema, thinning of the endometrial epithelium, and a reduction in gland number. Abnormal blood rheology was also observed, characterized by increased blood viscosity, enhanced erythrocyte aggregation, and accelerated erythrocyte sedimentation. Additionally, uterine artery blood flow resistance was elevated, while microcirculatory blood flow in the auricular and uterine surfaces was reduced. These pathological changes were ameliorated following LFWJD intervention, indicating that LFWJD improves hemorheological abnormalities and microcirculatory dysfunction.

The abnormalities in blood flow and microcirculation induced by cold stress are closely associated with vascular endothelial injury (Wang et al., 2024). ET-1, a potent vasoconstrictor peptide, and NO, a vasodilatory factor, are both synthesized by endothelial cells. The balance between ET-1 and NO is essential for maintaining vascular homeostasis (Kowalczyk et al., 2015). Excessive ET-1 production and reduced NO availability disrupt this balance, resulting in endothelial dysfunction. eNOS is a key enzyme responsible for NO synthesis, and its phosphorylated form represents the active state that enhances NO generation. Impaired eNOS activity leads to diminished NO production, further aggravating endothelial damage (Janaszak-Jasiecka et al., 2023). In addition, vWF and TM, which are secreted by endothelial cells, are critical markers of endothelial integrity, and their altered expression reflects endothelial dysfunction (Ozawa et al., 2024; de Melo et al., 2024).

The metabolites of LFWJD were identified by UHPLC-MS/MS. The major metabolites exhibited bioactivity in protecting vascular endothelial function and mitigating microvascular injury. Studies have shown that Albiflorin and Paeoniflorin ameliorate endothelial damage by inhibiting inflammation and inducible NO synthase (iNOS) gene expression (Zhang and Wei, 2020; Chen et al., 2015; Cao et al., 2025). Ferulic acid and its derivatives maintain NO homeostasis in endothelial cells, enhance eNOS expression, and thereby preserve endothelial function (Serreli et al., 2021). Liquiritin protects against hypoxia/reoxygenation-induced blood-brain barrier injury by suppressing ERS and reducing apoptosis (Li et al., 2021). Paeonol and Senkyunolide improve endothelial function in mice by alleviating ERS-mediated oxidative stress (Choy et al., 2017; Zhao et al., 2023). Cinnamaldehyde protects endothelial cells by suppressing high glucose-induced oxidative stress and apoptosis (Kim, 2022). In this study, we confirmed that cold stress led to elevated serum and uterine tissue levels of ET-1, vWF, and TM, alongside reduced NO levels and decreased p-eNOS/eNOS ratios in uterine tissues, indicating functional impairment of the uterine microvascular endothelium. LFWJD intervention reduced ET-1, TM, and vWF levels, restored NO and p-eNOS/eNOS, and improved endothelial ultrastructure, collectively demonstrating its ability to protect uterine microvascular endothelial function under cold stress. Given the polypharmacological characteristics of TCM formulas, identification of active metabolites based solely on UPLC-MS/MS may not fully capture all active constituents. Off-target effects and multi-component synergism are critical in mediating their pharmacological efficacy. In future studies, we will perform systematic separation and purification of LFWJD to identify metabolites with significant biological activities and observe their protective pharmacological effects on vascular injury.

Apoptosis is a major contributor to endothelial injury, and ERS is closely related to apoptosis. Inhibition of ERS has been shown to reduce apoptosis and alleviate vascular injury (Chen et al., 2024). Some studies have shown that endothelial dysfunction and apoptosis induced by obesity from high-calorie and high-cholesterol diets are strongly associated with ERS (Ye et al., 2013). Similarly, suppressing ERS reduces apoptosis of cardiac microvascular endothelial cells, and improves coronary microvascular dysfunction in patients with non-obstructive coronary artery disease (Li et al., 2019). However, limited research has addressed the role of ERS in vascular endothelial cell apoptosis under cold stress. Our findings demonstrated that cold stress induced significant apoptosis in rat uterine tissue and endothelial cells. Specifically, pro-apoptotic proteins Bax and cleaved-caspase3/caspase3 were upregulated, while the anti-apoptotic protein Bcl-2 was downregulated. Concurrently, the ERS markers GRP78 and CHOP were significantly elevated. Immunofluorescence analysis further revealed that CD34-labeled uterine microvascular endothelial cells exhibited increased Bax expression, reduced Bcl-2 expression, and elevated GRP78 and CHOP expression, collectively suggesting that cold stress promotes apoptosis of microvascular endothelial cells via ERS activation. Intervention with LFWJD reversed these changes, as evidenced by reduced GRP78, CHOP, and Bax expression, decreased cleaved-caspase3/caspase3, and increased Bcl-2 levels. These findings indicate that LFWJD alleviates cold-induced uterine microvascular injury, at least in part, by inhibiting ERS and thereby reducing endothelial cell apoptosis.

IRE1α is a critical protein of ERS that is widely expressed in various cell types (Raymundo et al., 2020). Among its downstream branches, the IRE1α/XBP1s and IRE1α/ASK1/JNK pathways are recognized as key signaling cascades mediating ERS-induced apoptosis (Shi et al., 2023). Under physiological conditions, GRP78 binds to IRE1α, maintaining it in an inactive state. However, during ERS, the accumulation of misfolded proteins sequesters GRP78, leading to IRE1α activation and subsequent stimulation of downstream pro-apoptotic signaling (Hetz et al., 2020). In our study, cold stress significantly increased the expression of XBP1s, p-IRE1α, p-JNK, and p-ASK1 proteins in uterine tissues, indicating activation of these ERS-related apoptotic pathways. Importantly, intervention with LFWJD reversed these changes, as evidenced by reduced expression of XBP1s, p-IRE1α, p-JNK, and p-ASK1. These findings suggest that LFWJD alleviates uterine microvascular injury by suppressing ERS and attenuating apoptosis, at least in part, through modulation of the IRE1α/XBP1s and IRE1α/ASK1/JNK signaling pathways.

4-PBA, a classical ERS inhibitor, significantly reduced the expression of GRP78, CHOP, p-IRE1α, p-JNK, and p-ASK, as well as the apoptosis rate and cleaved-caspase3/caspase-3 levels in rat uterine tissues. These findings provide direct evidence that suppression of ERS can attenuate apoptosis. On the one hand, this confirms that cold stress induces ERS-mediated apoptosis in rats; on the other hand, it supports the notion that the protective pharmacological effect of LFWJD on uterine microvascular endothelial function is at least partly attributable to its ability to alleviate ERS.

The composition and dosage of LFWJD used in this study were consistent with the Pharmacopoeia of the People’s Republic of China 2020 edition standards. The equivalent dose ratio between rats and humans is 6.3 (Nair et al., 2018). The dosage for experimental animals was calculated based on the body surface area of a 70-kg human. The research group previously observed the pharmacological effects of low-dose, medium-dose, and high-dose on uterine blood flow in cold-stressed rats. The results showed that the medium-dose and high-dose were significantly more effective than the low-dose, while no significant difference was observed between the medium-dose and high-dose (Xing et al., 2024; Guo, 2023). Therefore, in line with the 3Rs principle, this study selected low-dose (0.47 g/kg) and medium-dose (0.93 g/kg) as the administered doses.

## 5 Conclusion

**FIGURE 9:**
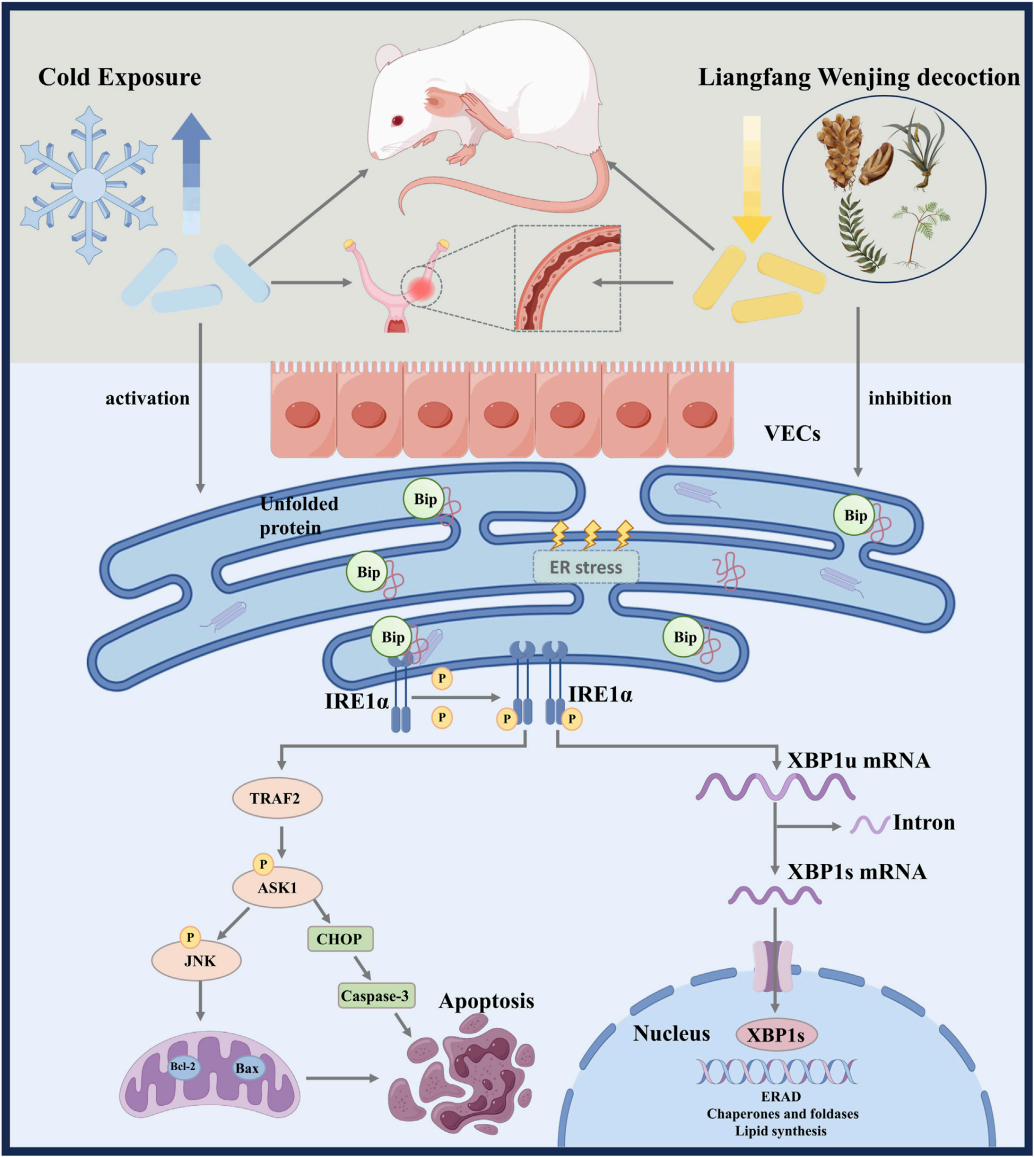
Schematic summary of LFWJD alleviates microvascular endothelial injury in cold-stressed rats.

Cold stress induces microvascular injury in uterine tissues. LFWJD may attenuate endoplasmic reticulum stress-mediated apoptosis to improve uterine microvascular injury in cold-stressed rats by regulating IRE1α/XBP1S and IRE1α/ASK1/JNK signaling pathways. This will provide an experimental basis for the potential clinical treatment of cold-induced gynecological disorders with LFWJD (Figure 9).

## Data Availability

The datasets presented in this study can be found in online repositories. The names of the repository/repositories and accession number(s) can be found in the article/Supplementary Material.

## Abbreviations

4-PBA, 4-phenyl butyric acid; ASK1, Apoptosis signal-regulated kinase 1; Bax, BCL2-associated X; Bcl-2, B-cell lymphoma 2; Caspase, Cysteinylaspartatespecificproteinase; CHOP, C/EBP homologous protein; ELISA, Enzyme-linked immunosorbent assay; eNOS, endothelial nitric oxide synthase; ER, Endoplasmic reticulum; ERS, Endoplasmic reticulum stress; ET-1, Endothelin-1; GRP78, Glucose-regulated protein 78; IRE1α, Inositol-dependent enzyme alpha; JNK, C-Jun amino-terminal kinase; LFWJD, Liangfang Wenjing decoction; NO, Nitric oxide; P-ASK1, Phosphorylated apoptosis signal-regulated kinase 1; P-eNOS, endothelial nitric oxide synthase; P-IRE1α, Phosphorylated inositol-dependent enzyme alpha; P-JNK, Phosphorylated amino-terminal protein kinase; TEM, Transmission electron microscopy; TM, Thrombomodulin; TUNEL, Terminal deoxynucleotidyl transferase-mediated nick-end labeling; UPR, Unfolded protein response; vWF, Vascular hemophilic factor; XBP1s, Shear X-box binding proteins.
