# Supplementary material for: Liangfang Wenjing decoction regulates endoplasmic reticulum stress-mediated apoptosis to alleviate uterine microvascular injury in cold-stressed rats
Source: Front Pharmacol. 2025 Sep 30;16:1649924. doi: 10.3389/fphar.2025.1649924 (PMC12517585; doi:10.3389/fphar.2025.1649924)
Supplement: Supplementary file 1 [file Table1.docx]

**Table S1** Main components of LFWJD identified by UHPLC Q-Orbitrap HRMS

| No. | Ingredient name | Retention time (min) | Molecular formula | Adduct | m/z observed | m/z theoretical | Δppm |
| --- | --- | --- | --- | --- | --- | --- | --- |
| 1 | Catechin | 6.93 | C_15_H_14_O_6_ | [M-H]- | 289.0719 | 353.0878 | 0.64 |
| 2 | Paeoniflorin | 9.77 | C_23_H_28_O_11_ | [M+FA-H]- | 525.1613 | 549.1614 | -0.05 |
| 3 | Ferulic acid | 10.68 | C_10_H_10_O_4_ | [M+H-H2O]+ | 177.0542 | 633.1814 | -1.97 |
| 4 | Liquiritin | 10.91 | C_21_H_22_O_9_ | [M-H]- | 417.1193 | 939.1109 | 0.57 |
| 5 | Senkyunolide H | 14.28 | C_12_H_16_O_4_ | [M+H-H2O]+ | 207.1012 | 131.0491 | -1.87 |
| 6 | Liquiritigenin | 14.51 | C_15_H_12_O_4_ | [M-H]- | 255.0660 | 235.1693 | -1.01 |
| 7 | Benzoylpaeoniflorin | 16.92 | C_30_H_32_O_12_ | [M+FA-H]- | 629.1879 | 167.0703 | 0.58 |
| 8 | Paeonol | 17.55 | C_9_H_10_O_3_ | [M+H]+ | 167.0698 | 329.2333 | -2.84 |
| 9 | Curcumenol | 20.03 | C_15_H_22_O_2_ | [M+H]+ | 235.1686 | 381.2060 | -2.81 |
