# Supplementary material for: Liangfang Wenjing decoction regulates endoplasmic reticulum stress-mediated apoptosis to alleviate uterine microvascular injury in cold-stressed rats
Source: Front Pharmacol. 2025 Sep 30;16:1649924. doi: 10.3389/fphar.2025.1649924 (PMC12517585; doi:10.3389/fphar.2025.1649924)
Supplement: Supplementary file 2 [file Table2.docx]

**Table S2** Blood hemorheological indexes in rats

| Group | Whole Blood (mpa. s) | | | Blood reduced viscosity (mpa. s) | | Erythrocyte aggregation | Casson viscosity | plasma viscosity |
| --- | --- | --- | --- | --- | --- | --- | --- | --- |
|  | High | Middle | Low | High | Low |  |  |  |
| Control | 4.03±0.05 | 5.08±0.05 | 16.42±0.16 | 5.75±0.29 | 34.10±0.23 | 4.03±0.04 | 3.42±0.05 | 1.28±0.04 |
| Model | 5.24±0.33^**^ | 7.43±0.77^*^ | 19.18±0.86^**^ | 6.79±0.36^**^ | 38.2±0.69^**^ | 5.32±0.13^**^ | 4.65±0.11^**^ | 1.62±0.06^**^ |
| LFWJD-D | 4.09±0.05^##^ | 5.28±0.05^#^ | 17.17±0.30^##^ | 5.89±0.17^##^ | 35.84±0.45^##^ | 4.12±0.06^##^ | 3.50±0.06^##^ | 1.37±0.05^##^ |
| LFWJD-H | 4.31±0.04^#^ | 6.00±0.15 | 18.04±0.33^##^ | 6.24±0.30^##^ | 37.33±0.41^##^ | 4.36±0.04^##^ | 3.88±0.08^##^ | 1.41±0.01^##^ |
| 4-PBA | 4.11±0.01^##^ | 5.30±0.03^#^ | 17.22±0.36^##^ | 5.89±0.14^##^ | 36.05±0.34^##^ | 4.15±0.03^##^ | 3.53±0.02^##^ | 1.38±0.06^##^ |

Notes: Blood hemorheological indexes in rats of each group. Blood hemorheology is an indication of the state of the blood. Compared with the control group, the hemorheological indices in the model group, whole blood reduced viscosity, and the erythrocyte aggregation index, were increased. The hemorheological indexes decreased after the intervention of LFWJD and 4-PBA. **p < 0.05, **p < 0.01* versus the control group; *#p < 0.05, ##p < 0.01* versus the model group *(*$\bar{x}$*±s, n=5)*.
